# Real clinical experience of inhaled esketamine in bipolar depression. A retrospective study

**DOI:** 10.1192/j.eurpsy.2025.1046

**Published:** 2025-08-26

**Authors:** S. Benavente López, A. Lara Fernández, E. Toro Carrasco, I. Pedrero Torrejon, A. Parra González, M. Mejia Quiterio, A. Viñas Arboleda, E. Losantos Ucha, E. Baca García

**Affiliations:** 1 Department of Psychiatry, Hospital Universitario Infanta Elena, Madrid, Spain

## Abstract

**Introduction:**

Bipolar depression is a severe type of depression that poses a clinical challenge when treating it, as it can often be resistant to treatment and there is also the possibility that when treated with antidepressants the patient may switch to a manic episode. In addition, there are few medications indicated for the depressive phase of bipolar depression, which reduces the therapeutic possibilities and makes the treatment of these patients even more complicated. Bipolar depression is associated with high rates of dysfunctionality and suicide, being a serious condition that can cause severe damage to the patient. Treatment with inhaled esketamine has been shown to be safe and effective in patients with bipolar depression.

The present study shows the response in a series of cases with bipolar depression treated with inhaled esketamine, showing their evolution through the Montgomery-Asberg Depression Rating Scale (MADRS) as main outcome.

**Objectives:**

The main objective of this study is to describe the use of inhaled esketamine in patients with bipolar depression in real clinical practice, using the MADRS as main outcome to show the evolution.

**Methods:**

Retrospective descriptive study with a sample selected by non-probabilistic consecutive sampling, retrospective type, in a time interval of 2 years. The patients selected were those who completed treatment with inhaled esketamine and had a diagnosis of bipolar depression at Hospital Universitario Infanta Elena. A descriptive analysis was performed. Mean and standard deviation were calculated for quantitative variables and N and percentage for categorical variables.

**Results:**

A total of 3 patients diagnosed with bipolar depression were included in the study (n: 3). Treatment with intranasal esketamine showed a clear improvement in the 3 patients with bipolar depression included in the study (100% response), greatly improving the MADRS score and restoring functionality to the patients. In addition, the treatment was well tolerated and no serious adverse effects occurred, with no switch to mania occurring in any of the patients.

**Conclusions:**

This study shows that inhaled esktemina may be a useful drug in the treatment of bipolar depression, showing high efficacy and good tolerability. No switch to mania was observed in any of the patients in this study. Longitudinal studies must be carried out to confirm this hypothesis.

**Disclosure of Interest:**

None Declared

